# Effects of Information Source Characteristics on Trust and Health Information Behaviours Among Individuals with Different Levels of Cyberchondria

**DOI:** 10.3390/bs16081359

**Published:** 2026-08-08

**Authors:** Yuanli Liu, Xiaoling Hu, Zeyang Yang

**Affiliations:** 1 Department of Psychology, School of Education, Soochow University, Suzhou 215123, China; 20244218052@stu.suda.edu.cn; 2 School of Chinese Language and Literature, Hubei University of Education, Wuhan 430205, China

**Keywords:** cyberchondria, information source characteristics, trust, health information behaviours

## Abstract

Online health information spreads quickly across different publishers and platforms. However, little is known about how information source characteristics, together with cyberchondria, influence individuals’ trust and intentions to share and verify. Using a 2 × 2 × 3 between-subject design, the present study investigated the effects of cyberchondria severity (high/low), publisher type (verified/unverified), and platform type (health app/search engine/social media) on individuals’ trust in health information and their intentions to share and verify that information. A total of 204 participants were included in this experiment. The results show that verified publishers and health apps can increase trust and the intention to share. Individuals with high cyberchondria show higher trust and intentions to share and verify, only when the publisher was unverified. The findings suggest that external authority (verification of accounts and professional content apps) can moderate the effects of cyberchondria or its related anxiety. The present study contributes to the literature on the determinants of online health behaviours by using an experimental design, with practical implications for platform management and online health information publishers.

## 1. Introduction

Cyberchondria refers to repetitive and excessive searching of online health information, which can intensify health anxiety (Starcevic & Berle, 2013). Online health information is produced by different publishers and distributed through different platforms, and its credibility is often difficult to evaluate (Duffy et al., 2020). Therefore, individuals might repetitively search for different sources on the internet and share them to obtain verification, which can lead to higher health anxiety (McMullan et al., 2019; Starcevic, 2017). Previous studies have found that information overload, uncertainty, and trust in online information are the key factors associated with cyberchondria (e.g., Laato et al., 2020; Weng et al., 2023). However, most studies have focused on individual difference factors related to cyberchondria, using self-report questionnaires, while experimental studies examining the interactive effects of individual and contextual factors on health information behaviours (e.g., sharing) remain limited. In particular, research on the impact of information source characteristics and an individual’s level of cyberchondria on health information behaviours is scarce. The present study aimed to explore the effects of information source characteristics (platform and publisher types) on information trust and health information behaviours (information sharing and verification) among individuals with different cyberchondria levels.

On some social media platforms, users can verify their accounts using their true identity by providing an ID card, a work status certificate, or other professional certificates. Such verification can enhance the credibility of the accounts and demonstrate the users’ influence (Deng et al., 2021; Lyu et al., 2021). Compared with verified users, unverified users may provide fewer credibility cues and may be associated with a higher proportion of inaccurate information (Deng et al., 2021; Kouzy et al., 2020). Unverified users lack credible identity verification, and the health information they publish is prone to being overlooked or doubted, which consequently forces information seekers to look for additional sources to verify its authenticity. Such repetitive searching behaviours appear to be a sign of cyberchondria. Therefore, it is necessary to explore how account verification influences individuals’ trust and health information behaviours, particularly among those with high and low cyberchondria.

Search engine indexing and ranking algorithms tend to display information about “serious and rare” diseases. When users search for common symptoms, the search results often lead to an escalation of health concerns (White & Horvitz, 2009). Using social media as a source of information will increase cyberchondria and information overload, leading to excessive public panic about health risks (Bala et al., 2021). Professional medical software or health information applications led by authoritative health institutions usually gain users’ trust more easily due to their high-quality information and strong credibility (Zheng et al., 2023). University students who obtain health information from less authoritative information platforms, such as newspapers/magazines, websites, and social media, reported significantly higher levels of cyberchondria than their peers who use government official websites (Bahadir & Dundar, 2024). In addition, research shows that the use of search engines can indirectly contribute to the emergence of cyberchondria by increasing the sense of information deficiency (Weng et al., 2023). A specific health application, “Dingxiang Doctor,” can significantly enhance users’ trust in online health information, thereby positively contributing to cyberchondria (Zheng et al., 2023). It is therefore necessary to compare whether different information sources or platforms influence individuals’ trust and health information behaviours, such as sharing.

Trust in information is a crucial factor shaping individuals’ online behaviour. Mayer et al. (1995) defined trust as an individual’s willingness to rely on others and to place oneself in an open state, in which one’s behaviours might be influenced by others’. Users’ trust in online information is often influenced by the perceived quality of the information, the credibility of the source, and the transmission channels (Cheung & Thadani, 2012). When dealing with information, anxious individuals often need to invest a significant amount of cognitive resources to assess the credibility of the information source in order to obtain content that is both trustworthy and meets their own needs (Ding & Yang, 2015). Research shows that online information trust has a significantly positive correlation with COVID-related internet-based health anxiety. Excessive trust in social media health information can exacerbate individual health anxiety (Laato et al., 2020). Zheng et al.’s (2023) study found that online information trust is positively correlated with cyberchondria.

Cyberchondria can not only affect an individual’s anxiety but also shape their health information behaviours, such as sharing and verification. A recent study shows that individuals with high levels of cyberchondria tend to share health information even when it lacks verification (Xiao et al., 2025). Information overload further reinforces this tendency (Laato et al., 2020). Meanwhile, cyberchondria was found to be linked with the intention of verifying online health information. Individuals might repetitively check different sources to reduce their sense of uncertainty (Sun, 2022). However, such repetitive verification behaviours can increase anxiety, which might lead to a vicious loop of searching and doubting (McMullan et al., 2019). These findings suggest that cyberchondria can affect individuals’ sharing and verification behaviours. However, it remains unknown whether such a link varies across information source characteristics, such as publisher identity and platform type.

Drawing on uncertainty reduction theory and uncertainty management perspectives, individuals may seek additional information when they face ambiguous health-related information (Berger & Calabrese, 1975; Brashers, 2001). For individuals with higher cyberchondria, such uncertainty may be linked to repeated searching, sharing, or verification as ways to obtain reassurance or reduce uncertainty. In this process, sharing may help individuals obtain feedback or confirmation from others, whereas verification may reflect further checking across sources. However, these behaviours may vary according to source credibility cues. Verified publishers and professional health platforms may reduce uncertainty by providing external signals of credibility, whereas unverified publishers or less authoritative platforms may require more cognitive resources to evaluate the information (Sweller, 1988). Therefore, publisher identity and platform type may moderate the associations between cyberchondria and health information sharing and verification.

Although existing studies have examined the links among cyberchondria, information trust, and online health information behaviours, little attention has been paid to the interactive effects of individual differences and information source characteristics on health information behaviours within a single framework. In particular, it remains unclear how publisher identity and platform type jointly influence trust, sharing, and verification across different levels of cyberchondria. The present study aims to fill this gap by examining: (1) how cyberchondria relates to trust, sharing, and verification of health information and (2) how publisher identity and platform type shape these relationships. Several hypotheses can be proposed as follows:

Information trust:

**H1a.**  *Higher cyberchondria is associated with higher trust in health information.*

**H1b.**  *Information from verified users leads to higher trust than information from unverified users.*

**H1c.**  *Health apps lead to higher trust than search engines and social media.*

**H1d.**  *Cyberchondria moderates the effects of publisher identity and platform type on trust.*

Information sharing:

**H2a.**  *Higher cyberchondria is associated with a stronger willingness to share health information.*

**H2b.**  *Information from verified users leads to higher sharing intention than information from non-verified users.*

**H2c.**  *Health apps lead to higher sharing intention than search engines and social media.*

**H2d.**  *Cyberchondria moderates the effects of publisher identity and platform type on sharing intention.*

Information verification:

**H3a.**  *Higher cyberchondria is associated with a stronger willingness to verify health information.*

**H3b.**  *Information from verified users leads to higher verification intention than information from non-verified users.*

**H3c.**  *Health apps lead to higher verification intention than search engines and social media.*

**H3d.**  *Cyberchondria moderates the effects of publisher identity and platform type on verification intention.*

## 2. Materials and Methods

### 2.1. Participants

The required sample size was calculated using G*Power 3.1. A three-way ANOVA was specified, with a significance level of α = 0.05 and a medium effect size f = 0.25. To achieve a statistical power of 0.80 (Faul et al., 2007), a minimum total sample size of 158 participants was needed. The target source population was adult Chinese internet users. The minimum sample size was based on the planned three-way ANOVA and the assumed medium effect size. Through the online survey platform Credamo, 204 participants were recruited, including 70 males and 134 females, with a mean age of 31.79 ± 8.40 years. After providing informed consent, all participants completed the questionnaire and were informed about the anonymity of the study and data confidentiality measures. Participants received compensation based on the quality of their responses after completing the survey. The study was approved by the Ethics Committee of the authors’ university and strictly adhered to the ethical principles outlined in the Declaration of Helsinki.

### 2.2. Experimental Materials

This study primarily utilises information related to lung cancer as the experimental material. Lung cancer, due to its highly aggressive and easily metastasising biological characteristics, is widely recognised as the leading cause of global cancer-related deaths (Lemjabbar-Alaoui et al., 2015). According to global cancer statistics, China, bearing one of the heaviest burdens of lung cancer, accounts for approximately one-third of both the new cases and deaths worldwide (Long et al., 2023). Given its high incidence, high mortality, and broad public awareness, lung cancer readily triggers public health concerns and online information-seeking behaviours. Therefore, this study employs lung cancer-related symptoms as the experimental scenario for investigating cyberchondria.

As the independent variables, we manipulated the “information publisher” (Verified user/Unverified user) and the “information platform” (search engine: Baidu/health app: Dingxiang Doctor/social media: Xiaohongshu). The materials for each condition included screenshots of the platform interface and the specific information content, simulating a real online information browsing environment. In the present study, “manipulated materials” referred to the experimental screenshots shown to participants. Each screenshot contained the same lung cancer-related health information but differed in the publisher identity and platform type displayed. Thus, participants were exposed to a simulated online health information post rather than an intervention or educational material. Materials attributed to “verified users” were labelled as content from authenticated accounts with public attention, authenticity, reputation, and activity (e.g., marked with a “V” badge). Materials for “unverified users” corresponded to content posted by ordinary, unauthenticated users. All materials revolved around the common health theme of “persistent cough, chest pain, shortness of breath accompanied by weight loss,” ensuring thematic consistency. Variations were strictly limited to the identity of the information publisher and the platform type, thereby controlling for potential confounding effects of content variation on the study results.

### 2.3. Research Procedure

A 2 (cyberchondria: high/low) × 2 (information publisher: verified/unverified user) × 3 (information platform: search engine/health app/social media) between-subject design was used. The dependent variables included information trust, information sharing intention, and information verification intention. Before the experiment began, participants were informed that “this experiment is conducted anonymously, and all collected data will be used solely for academic research purposes,” and they provided informed consent online. First, participants were presented with a set of instructions describing a scenario where they experienced suspected lung cancer-related symptoms (e.g., persistent cough, chest pain) and proceeded to search for information online. Subsequently, each participant was randomly assigned to view one of the six sets of experimental materials, which simulated a screenshot of health information posted by either a verified or an unverified user on a specific platform (Baidu, Dingxiang Doctor, or Xiaohongshu). This hypothetical scenario design was used to ensure that the health information content was held constant across conditions while publisher identity and platform type were experimentally manipulated. Such control was necessary for isolating the effects of information source characteristics on trust, sharing intention, and verification intention. Immediately after viewing the material, participants were required to complete a series of measures: they first answered a comprehension check question regarding the type of information publisher and the platform. Following this, they sequentially completed single-item measures assessing their sharing intention, verification intention, and trust in the content of the presented image. Finally, participants filled out the Cyberchondria Severity Scale and provided demographic information.

### 2.4. Research Instruments

#### 2.4.1. Cyberchondria

The short-form version of the Cyberchondria Severity Scale (CSS-12), developed by McElroy et al. (2019), was used to measure participants’ levels of cyberchondria. The scale consists of 12 items rated on a 5-point Likert scale (1 = strongly disagree, 5 = strongly agree). It comprises four dimensions: Compulsion, Distress, Excessiveness, and Reassurance, with each dimension measured using three items. A higher total score indicates a higher level of cyberchondria. In the current study, the scale demonstrated good reliability (Cronbach’s α = 0.901; *M* = 3.38, *SD* = 0.73).

#### 2.4.2. Information Trust

A single item adapted from Jiang et al. (2021) was used to assess participants’ trust in the health information: “I believe the health information in the figure above is trustworthy” (1 = strongly disagree, 5 = strongly agree; *M* = 3.42, *SD* = 0.96).

#### 2.4.3. Information Sharing Intention

A single item based on Wang (2022) was used to assess participants’ intention to share health information: “I will share the above health information with people I care about” (1 = strongly disagree, 5 = strongly agree; *M* = 3.67, *SD* = 1.36).

#### 2.4.4. Information Verification Intention

A single item based on Lim and Hong (2025) was used to assess participants’ intention to verify health information: “I want to continue searching for related health information at present” (1 = strongly disagree, 5 = strongly agree; *M* = 4.21, *SD* = 0.98).

### 2.5. Data Analysis

The data were analysed using SPSS version 27. First, descriptive statistical analyses were performed on all data. Second, to validate the effectiveness of the cyberchondria grouping, an independent samples *t*-test was conducted to examine whether the difference between the high cyberchondria group and the low cyberchondria group was significant. Furthermore, to examine the effects of cyberchondria grouping, information publisher, and information platform on the dependent variables, three separate factorial ANOVAs were performed with information trust, information sharing intention, and information verification intention as the dependent variables, respectively.

## 3. Results

### 3.1. Descriptive Statistics

Table 1 presents the demographic information of the 204 participants. The majority were female, accounting for 65.7% (134/204). Ages ranged from 19 to 65 years (*M* = 31.79, *SD* = 8.40). Overall, the participants’ educational level was relatively high, with most (69.6%) holding a bachelor’s degree. No participants had an educational level of junior high school or below.

### 3.2. Manipulation Checks

To verify the effectiveness of the experimental manipulations, manipulation checks were first conducted. An independent samples *t*-test was performed to check the manipulation of cyberchondria grouping. The results indicated a significant difference between the two groups (*t* = −21.00, *p* < 0.001). Participants in the high cyberchondria group (*M* = 3.98, *SD* = 0.31) scored significantly higher than those in the low cyberchondria group (*M* = 2.78, *SD* = 0.49).

To test the effectiveness of the manipulations for “information publisher” and “information platform,” all participants were required to answer two direct manipulation check questions after reading the experimental materials: “Do you believe the content in the above picture is from the Baidu (Dingxiang Doctor/Xiaohongshu) platform? Yes or No”; and “Do you believe the information publisher in the above picture is a verified user or an unverified user? Verified user or Unverified user.” The manipulation check results showed that the accuracy rate for participants’ judgments regarding the information platform and the verification status of the information publisher was 100%. Therefore, the experimental manipulations for both the information publisher and the information platform were effective.

### 3.3. Analysis of Variance Results

#### 3.3.1. Effects of Cyberchondria, Information Publishers, and Information Platforms on Information Trust

A three-way ANOVA was conducted with cyberchondria, information publisher, and information platform as independent variables. The results revealed a significant main effect of cyberchondria on information trust (*F* (1, 192) = 16.781, *p* < 0.001, *η*^2^*p* = 0.08). Participants in the high cyberchondria group (*M* = 3.68, *SD* = 0.80) reported a higher level of trust in the health information than those in the low cyberchondria group (*M* = 3.16, *SD* = 1.03). H1a was supported.

The main effect of information publisher was also significant (*F* (1, 192) = 76.127, *p* < 0.001, *η*^2^*p* = 0.28). Participants showed a higher level of trust in health information posted by “verified users” (*M* = 3.90, *SD* = 0.61) compared to that posted by “unverified users” (*M* = 2.93, *SD* = 1.00). H1b was supported.

The main effect of the information platform on information trust was also significant (*F* (2, 192) = 3.687, *p* < 0.05, *η*^2^*p* = 0.04). Further post hoc comparisons revealed that participants’ trust in health information from the “search engine” group (*M* = 3.32, *SD* = 1.04) was significantly lower (*p* < 0.05) than that from the “health app” group (*M* = 3.63, *SD* = 0.81). Trust in health information from the “health app” group was significantly higher (*p* < 0.05) than that from the “social media” group (*M* = 3.29, *SD* = 0.98). No significant difference in trust (*p* > 0.05) was found between the “search engine” group and the “social media” group. H1c was supported.

**Figure 1:**
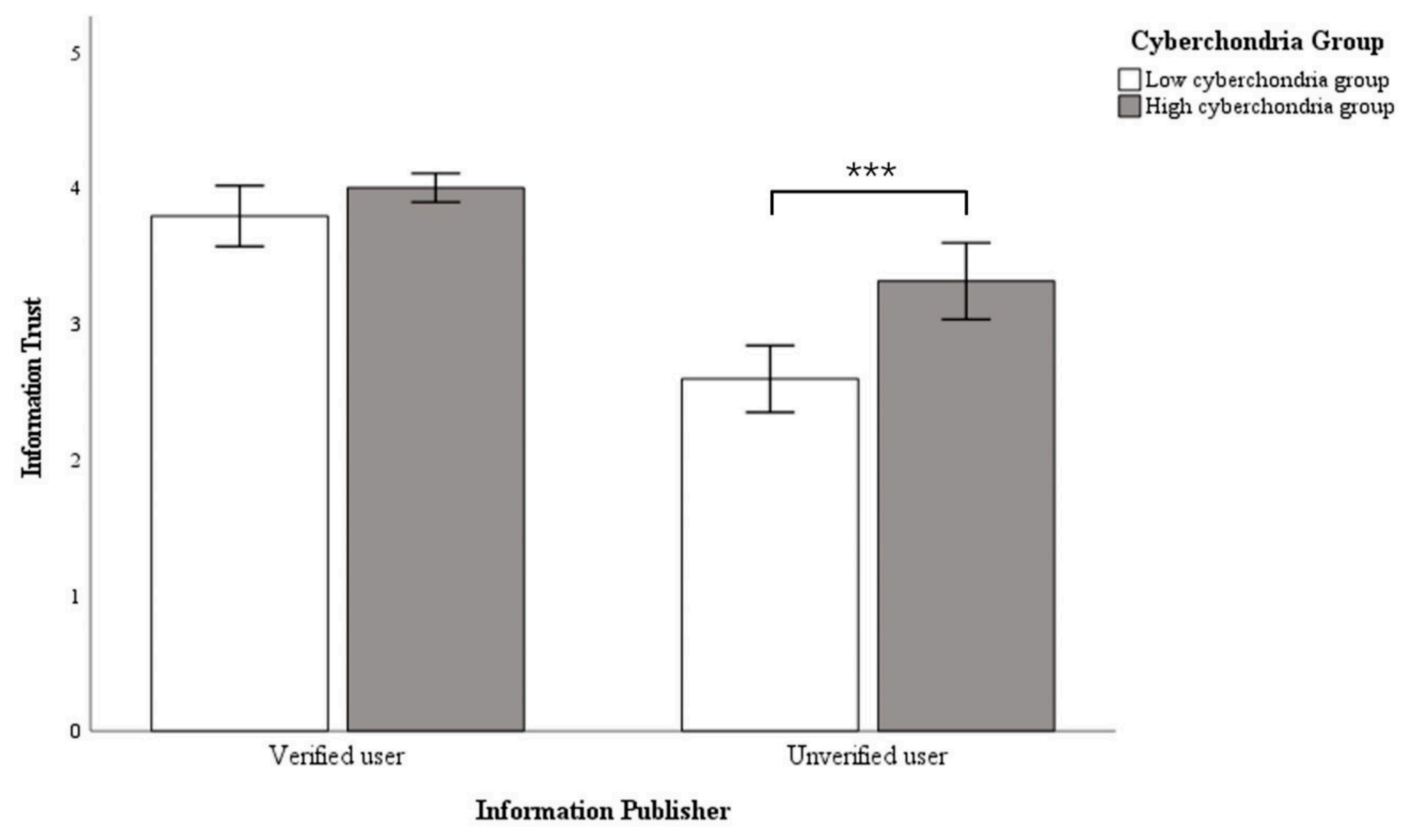
Interaction between high–low cyberchondria and information publisher on information trust. Note. *** *p* < 0.001.

Furthermore, the interaction between cyberchondria and information publisher was significant (*F* (1, 192) = 4.448, *p* < 0.05, *η*^2^*p* = 0.02). Simple effects analysis showed that under the “verified user” condition, the level of trust in health information did not differ significantly between the cyberchondria groups (*p* > 0.05). However, under the “unverified user” condition, the “high cyberchondria” group (*M* = 3.28, *SD* = 0.11) reported a significantly higher level of trust in the health information (*p* < 0.001) than the “low cyberchondria” group (*M* = 2.60, *SD* = 0.11). See Figure 1 for the interaction.

The interaction between cyberchondria and information platform (*F* (2, 192) = 2.004, *p* > 0.05, *η*^2^*p* = 0.02), as well as between information platform and information publisher (*F* (2, 192) = 1.587, *p* > 0.05, *η*^2^*p* = 0.02), on information trust were not significant. H1d was partially supported.

#### 3.3.2. Effects of Cyberchondria, Information Publishers, and Information Platforms on Information Sharing Intention

Analysis of variance results revealed a significant main effect of cyberchondria on information sharing intention (*F* (1, 192) = 14.321, *p* < 0.001, *η*^2^*p* = 0.07). Participants in the “high cyberchondria” group (*M* = 4.03, *SD* = 1.16) demonstrated a significantly higher intention to share the health information than those in the “low cyberchondria” group (*M* = 3.31, *SD* = 1.46). H2a was supported.

The main effect of information publisher on information sharing intention was also significant (*F* (1, 192) = 67.983, *p* < 0.001, *η*^2^*p* = 0.26). Participants exhibited a stronger sharing intention for health information posted by “verified users” (*M* = 4.33, *SD* = 0.85) compared to information posted by “unverified users” (*M* = 3.01, *SD* = 1.45). H2b was supported.

The main effect of the information platform on information sharing intention was also significant (*F* (2, 192) = 4.290, *p* < 0.05, *η*^2^*p* = 0.04). Further post hoc comparisons revealed that the sharing intention in the “search engine” group (*M* = 3.59, *SD* = 1.42) was significantly lower (*p* < 0.05) than that in the “health app” group (*M* = 4.00, *SD* = 1.28). The sharing intention in the “health app” group was significantly higher (*p* < 0.05) than that in the “social media” group (*M* = 3.43, *SD* = 1.33). No significant difference in sharing intention (*p* > 0.05) was found between the “search engine” group and the “social media” group. H2c was supported.

**Figure 2:**
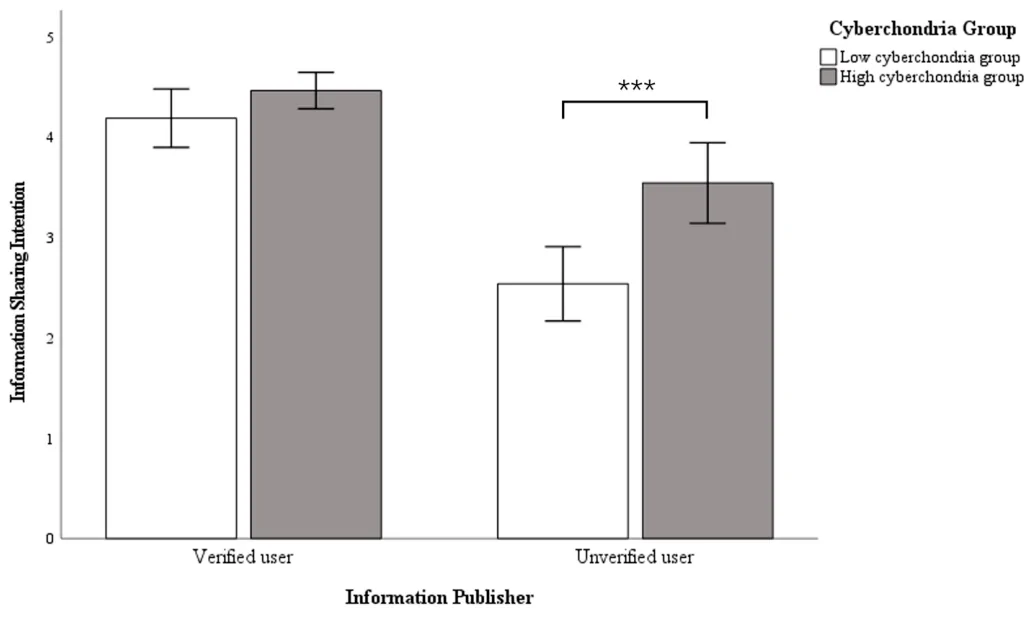
Interaction between high–low cyberchondria and information publisher on information sharing intention. Note. *** *p* < 0.001.

Furthermore, the interaction between cyberchondria and information publisher was significant (*F* (1, 192) = 4.787, *p* < 0.05, *η*^2^*p* = 0.02). Further simple effects analysis indicated that under the “verified user” condition, there was no significant difference in sharing intention between the cyberchondria groups (*p* > 0.05). However, under the “unverified user” condition, the “high cyberchondria” group (*M* = 3.50, *SD* = 0.16) exhibited a significantly higher sharing intention for the health information (*p* < 0.001) than the “low cyberchondria” group (*M* = 2.55, *SD* = 0.15). See Figure 2 for the interaction.

The interaction between cyberchondria and information platform (*F* (2, 192) = 1.360, *p* > 0.05, *η*^2^*p* = 0.01), as well as information platform and information publisher (*F* (2, 192) = 0.606, *p* > 0.05, *η*^2^*p* = 0.01), on information sharing intention were not significant. H2d was partially supported.

#### 3.3.3. Effects of Cyberchondria, Information Publishers, and Information Platforms on Information Verification Intention

The results of the analysis of variance revealed a significant main effect of cyberchondria on information verification intention (*F* (1, 192) = 23.400, *p* < 0.001, *η*^2^*p* = 0.11). Participants in the “high cyberchondria” group (*M* = 4.52, *SD* = 0.64) demonstrated a higher verification intention for the health information than those in the “low cyberchondria” group (*M* = 3.89, *SD* = 1.15). H3a was supported.

The main effect of information publisher on information verification intention was also significant (*F* (1, 192) = 28.480, *p* < 0.001, *η*^2^*p* = 0.13). Participants exhibited a stronger verification intention for health information posted by “verified users” (*M* = 4.55, *SD* = 0.68) compared to information posted by “unverified users” (*M* = 3.86, *SD* = 1.11). H3b was supported.

The main effect of the information platform on information verification intention was not significant (*F* (2, 192) = 0.735, *p* > 0.05, *η*^2^*p* = 0.01). H3c was not supported.

**Figure 3:**
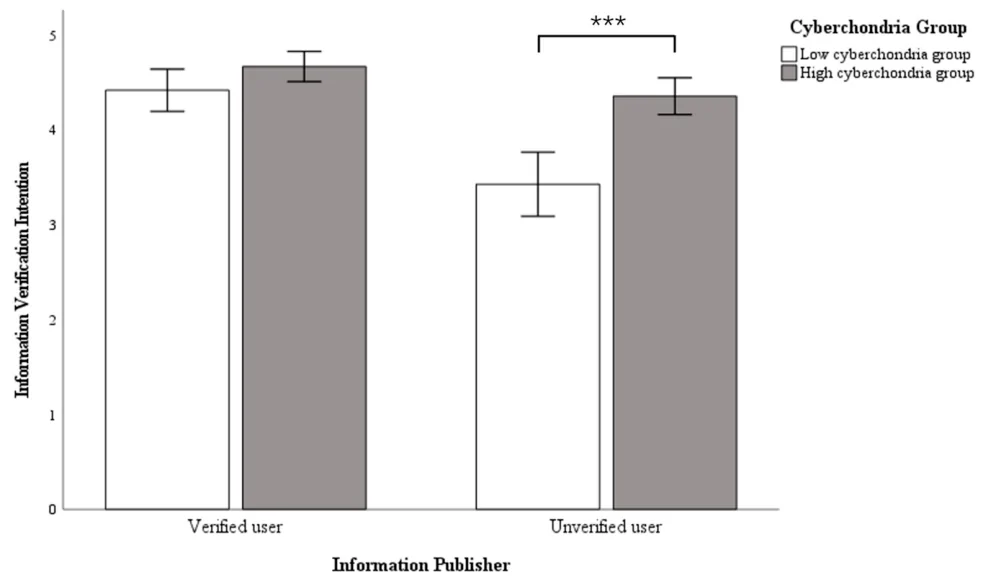
Interaction between high–low cyberchondria and information publisher on information verification intention. Note. *** *p* < 0.001.

Furthermore, the analysis of interaction revealed a significant interaction between cyberchondria and information publisher on information verification intention (*F* (1, 192) = 7.561, *p* < 0.05, *η*^2^*p* = 0.04). Further simple effects analysis revealed that under the “verified user” condition, there was no significant difference in verification intention between the cyberchondria groups (*p* > 0.05). However, under the “unverified user” condition, the “high cyberchondria” group (*M* = 4.35, *SD* = 0.12) exhibited a significantly higher verification intention for the health information (*p* < 0.001) than the “low cyberchondria” group (*M* = 3.41, *SD* = 0.13). See Figure 3 for the interaction.

**Figure 4:**
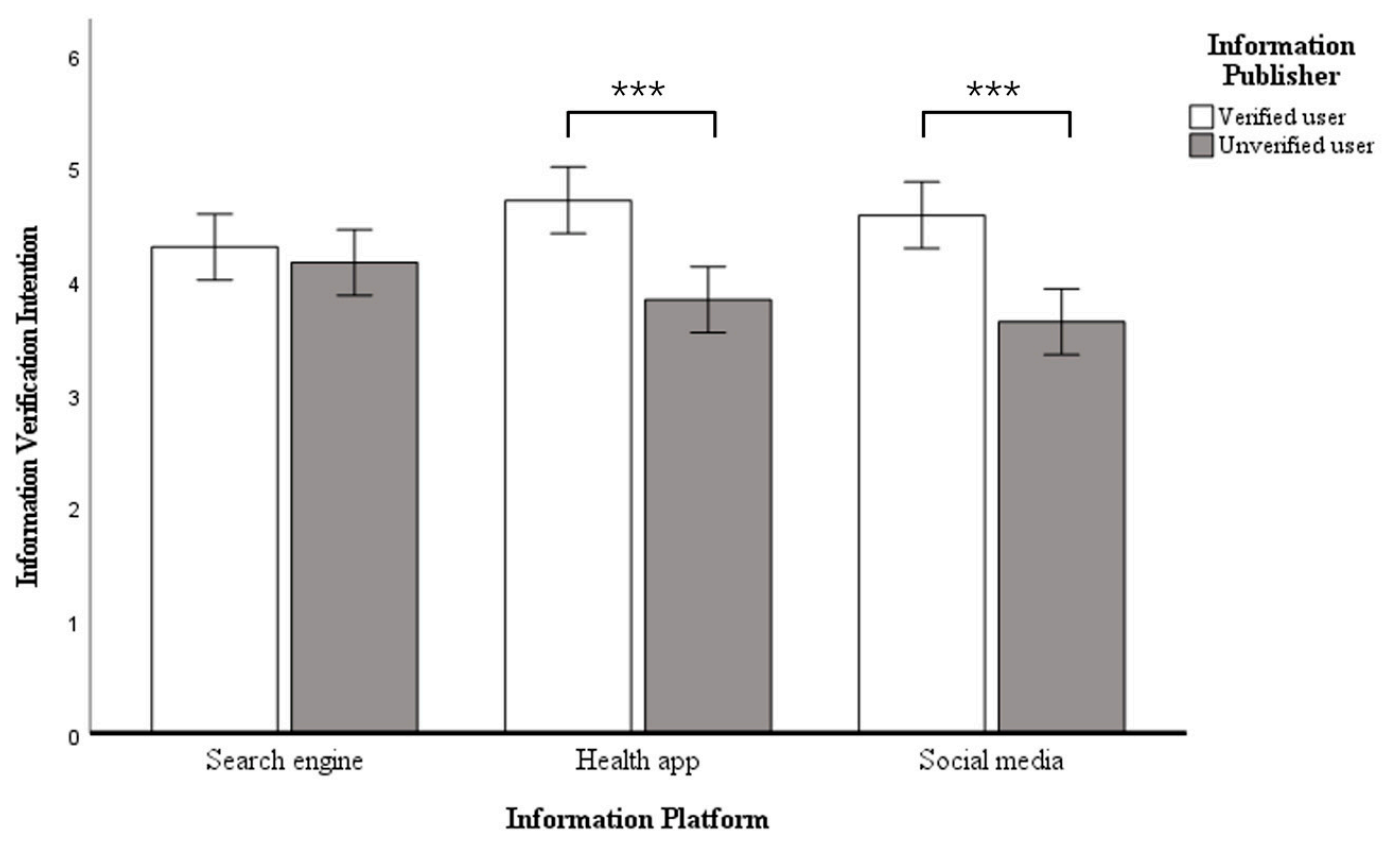
Interaction between information platform and information publisher on information verification intention. Note. *** *p* < 0.001.

Furthermore, a significant interaction was found between information platform and information publisher on information verification intention (*F* (2, 192) = 4.202, *p* < 0.05, *η*^2^*p* = 0.04). Further simple effects analysis revealed that under the “search engine” condition, no significant difference was found for verification between the “verified user” (*M* = 4.32, *SD* = 0.15) and the “unverified user” (*M* = 4.17, *SD* = 0.15). Under the “health app” condition, the “verified user” (*M* = 4.75, *SD* = 0.15) generated a significantly higher verification intention (*p* < 0.001) than the “unverified user” (*M* = 3.83, *SD* = 0.15). Similarly, under the “social media” condition, the “verified user” (*M* = 4.55, *SD* = 0.15) generated a significantly higher verification intention (*p* < 0.001) than the “unverified user” (*M* = 3.68, *SD* = 0.15). See Figure 4 for the interaction.

The interaction between cyberchondria and the information platform on information verification intention was not significant (*F* (2, 192) = 1.360, *p* > 0.05, *η*^2^*p* = 0.01). H3d was partially supported.

## 4. Discussion

### 4.1. Summary of the Findings

The present study investigated the effects of cyberchondria, information publisher type, and platform type on individuals’ health information trust, sharing intention, and verification intention. Interaction effects were detected. Information from verified users was more trusted and led to higher sharing and verification intention. Individuals with higher cyberchondria demonstrated higher trust, sharing intention, and verification intention toward the given materials, but only in the unverified publisher condition. Health apps were trusted more and generated higher sharing intention than search engines and social media.

### 4.2. Relationship with Previous Findings and Implications

The results of the present study show that information source characteristics (publisher and platform types) can affect trust and health information behaviours. First, health information posted by verified publishers was more trusted than that from unverified users. Similar to previous studies, individuals appear to place greater trust in content generated by verified accounts with more reliable identification (e.g., Cheung & Thadani, 2012). In addition to publisher type, platform type also influences information trust. The present study revealed that health information is more trusted when posted on health applications than on social media or search engines, a finding consistent with previous studies (Zheng et al., 2023). Such results show that individuals not only care about the publisher’s identity but also consider where the information comes from and perceive health applications as a more reliable source. They might perceive online health applications as a professional platform rather than search engines or social media. However, the reasons behind such results are not clear, and the participants’ qualitative or narrative perceptions are needed. The non-significant interaction between platform type and publisher identity on trust may indicate that these two source cues operate relatively independently. In other words, verified identity and professional platform context may each increase perceived credibility, but their combined effect may not necessarily be stronger than their separate effects.

Furthermore, the sharing and verification intentions for health information can differ across publishers and platforms. The present study shows that individuals would like to share the health information provided by verified publishers and by more professional health applications. It suggests, together with previous studies, that verification of a publisher can be a sign of certainty and credibility (Cunningham & Johnson, 2016; Deng et al., 2021; Johnson et al., 2015; Lyu et al., 2021). One possible explanation is that verified publishers and health applications provide external credibility cues, which may make individuals more willing to share the information. Such sharing may also be related to social feedback or impression management concerns. Health information sharing may also have broader social consequences for individuals with cyberchondria. When health information is shared within social networks, online communities, or interpersonal relationships, it may not only disseminate health-related concerns but may also amplify anxiety through repeated exposure and social discussion. Such exposure could influence how individuals perceive and interpret bodily symptoms, potentially reinforcing maladaptive health beliefs or repeated checking behaviours. However, these social processes were not directly measured in the present study. Future studies could examine how health information sharing affects symptom perception and anxiety in social contexts. However, the present study did not directly measure social capital or fear of social rebuff, and these explanations should therefore be interpreted cautiously. Moreover, the verification intention can be influenced by publisher type, and this effect holds only for health application and social media. In line with recent studies on health information verification and online misinformation, individuals may verify health information when they perceive uncertainty or when they wish to evaluate the credibility of online content (Sun, 2022; Lim & Hong, 2025). The present findings suggest that verified publishers may encourage verification intention by increasing perceived credibility, whereas the effect of platform type on verification intention was less clear. Verification of information from trusted sources may reflect continued engagement with credible health information rather than only defensive checking. Such verification intention might represent an individual’s trust and social identification with authoritative health information (Zheng et al., 2023) and their triangulation of health information in their social relationships. However, the non-significant effect of platform type on verification intention suggests that verification may not depend only on whether the information appears on a search engine, health application, or social media platform. Instead, verification may reflect a more general tendency to reduce uncertainty or seek reassurance when individuals encounter health-related information.

The present study shows that the severity of cyberchondria can influence an individual’s trust and health information behaviours. Individuals with higher cyberchondria are more likely to trust, share, and verify online health information than those with lower cyberchondria, which supports previous studies (e.g., Sun, 2022). Such behaviours might reflect their high health anxiety about symptoms, developed in the vicious circle of searching and being anxious (McMullan et al., 2019). However, this effect was observed only in the unverified publisher condition, which is consistent with previous findings (Xiao et al., 2025). In other words, the verification of a publisher account serves as a crucial external trigger of authority and security, reducing uncertainty about the information. For the high cyberchondria group, such authority might moderate their anxiety and related vigilance. Therefore, the publisher’s verification can be considered a sign of credible information, which can mitigate the effects of individual differences on information perception.

The present study has potential theoretical and practical implications. The results show that platform type is a key cue affecting an individual’s trust in online health information and sharing intention, beyond the verification state of publishers. It could enhance the understanding of the role of stimuli and situations as noted in the Cognitive-Behavioural Model of Hypochondriasis (Salkovskis et al., 2003). The impact of information source characteristics (i.e., the situations) on trust and health information behaviours could be multi-layered. Furthermore, cyberchondria and the publisher interactively affected trust. Such a finding indicates that trust in health information is not solely determined by individual differences or account verification. In practice, the findings may provide several suggestions for online health information management and communication. For platform managers, clearer verification labels and more visible source credibility cues may help users evaluate health information more easily. The findings also suggest that health information posted by unverified accounts should be monitored carefully, as individuals with higher cyberchondria may still trust, share, or verify such information. For health communicators, professional health applications or other authoritative channels may be more suitable for disseminating health information. For clinicians and mental health practitioners, it may be useful to encourage individuals with cyberchondria to evaluate source credibility before repeatedly searching, sharing, or verifying health information online.

### 4.3. Limitations and Future Studies

The present study has several limitations. This study relied solely on hypothetical scenarios and self-reported sharing or verification intentions, both of which may be biassed. Future studies can design real-world health information-searching tasks and record participants’ actual behaviours, such as the times they repeat searches or the websites they visit. Furthermore, the sample in the present study might not represent the larger population. In addition, participants received monetary compensation after completing the survey. Compensation may introduce selection bias by attracting individuals who are more motivated by financial incentives. This may affect the representativeness of the sample and the quality or reliability of responses. Future studies could recruit a more representative sample and improve the generalizability of the results. Moreover, the present study did not measure the participants’ personal characteristics, such as personality. It would be necessary for future studies to measure participants’ personality and explore whether it moderates the effects. The present study included only three platforms, which may not represent the true internet environment. Future studies could compare more platform types and explore which platform is the most trusted. In addition, the present study did not directly measure social capital, fear of social rebuff, perceived uncertainty, or other psychosocial mechanisms discussed above. Future studies could directly measure these psychosocial processes to examine whether they account for the associations between cyberchondria and health information sharing or verification. The present study used lung cancer as the only disease context. Although lung cancer is a highly salient health topic, the findings may not be fully generalisable to other health conditions with different levels of severity, familiarity, or perceived controllability. Future studies could examine whether similar effects appear in other disease contexts. Future studies could also conduct similar experiments in different contexts and investigate the potential cross-cultural or cross-national differences.

Furthermore, the present study used single items to assess participants’ trust, sharing, and verification intentions. Although single-item measures can be efficient in experimental designs with multiple conditions, they may provide limited reliability and restricted content coverage compared with multi-item scales. Consequently, the precision of the estimated effects on these dependent variables may be constrained. Future studies could therefore adopt validated multi-item scales of information trust, sharing intention, and verification intention to improve measurement reliability and validity.

In addition, cyberchondria was dichotomised using a median split approach to form high and low groups for the factorial design. However, median splits can reduce statistical power and obscure finer-grained individual differences along a continuous distribution. It appears that treating cyberchondria as a continuous moderator would allow for a more sensitive test of whether its associations with trust and health information behaviours vary as a function of publisher identity and platform type. Future studies could therefore retain the continuous CSS-12 scores and examine continuous moderation analyses (e.g., moderated regression or PROCESS-based models), which may provide a more nuanced understanding of these interactive effects.

## 5. Conclusions

The present study found that verified publishers and health application platforms can increase trust in health information and intentions to share. Individuals with high cyberchondria demonstrated higher trust, sharing and verification intentions only towards those from unverified publishers. Platform characteristics, particularly verification badges, can moderate the effects of cyberchondria on health information behaviours. The findings provide a deeper understanding of when and by whom online health information is trusted, shared, or further verified.

## Figures and Tables

**Table 1:** Demographic characteristics of the sample.

Variable	Category	Frequency	Percentage (%)
Age	18–30	93	45.6
	31–45	95	46.6
	46–60	15	7.4
	>60	1	0.5
Gender	Male	70	34.3
	Female	134	65.7
Education	High school/junior college	26	12.7
	Undergraduate	142	69.6
	Postgraduate and above	36	17.6

## Data Availability

The data are not publicly available due to privacy concerns.
